# Comparison of chemical-induced temporomandibular osteoarthritis rat models (monosodium iodoacetate versus collagenase type II) for the study of prolonged drug delivery systems

**DOI:** 10.1371/journal.pone.0281135

**Published:** 2023-01-31

**Authors:** Florent Barry, Feng Chai, Henry Chijcheapaza-Flores, Maria José Garcia-Fernandez, Nicolas Blanchemain, Romain Nicot

**Affiliations:** 1 INSERM, CHU Lille, U1008 - Controlled Drug Delivery Systems and Biomaterials, University of Lille, Lille, France; 2 INSERM, CHU Lille, Department of Oral and Maxillofacial Surgery, University of Lille, Lille, France; University of Catanzaro, ITALY

## Abstract

**Objective:**

To compare two agents that can induce a rat model of temporomandibular joint osteoarthritis (TMJOA) by chemical induction: monosodium iodoacetate (MIA) and collagenase type 2 (Col-2). We wished to ascertain the best agent for assessing drug-delivery systems (DDSs).

**Method:**

Male Wistar rats underwent intra-articular injection with MIA or Col-2. They were manipulated for 30 days. The head withdrawal threshold (HWT), immunohistological assessment, and positron emission tomography (PET) were used to evaluate the relevance of our models.

**Results:**

For both the MIA and Col-2 groups, pain persisted for 30 days after injection. Change in the HWT showed that Col-2 elicited a strong action initially that decreased progressively. MIA had a constant action upon pain behavior. Histology of TMJ tissue from both groups showed progressive degradation of TMJ components.

**Conclusions:**

MIA and Col-2 induced orofacial pain by their local chemical action on TMJs. However, based on a prolonged and greater sustained effect on the pain threshold, persistent histological changes, and imaging results, MIA appeared to be more suitable for creation of a rat model of TMJOA for the study of DDSs.

## Introduction

Temporomandibular disorders (TMDs) are myoarthropathies of the manducatory system. They affect ~31% of adults and ~11% of children [1]. TMDs are responsible for most cases of chronic pain in oral and maxillofacial regions. They have a significant impact on healthcare costs and the quality of life (QoL) of patients, and may complicate an existing systemic disease, such as multiple sclerosis [2]. Conventionally, TMDs are divided into two groups according to their manifestation: muscular or articular [3]. Among masticatory muscle disorders are muscle pain, contracture, and hypertrophy. Temporomandibular joint (TMJ) disorders are divided into five separate entities: joint paint, diseases or disorders; congenital disorders; and fractures. The major concern in joint disease TMDs is temporomandibular joint osteoarthritis (TMJOA). TMJOA is a chronic arthritic disease characterized by excessive degradation of bone and cartilage, reduction of the amount of synovial fluid, and persistent synovial inflammation [4, 5]. TMJOA can lead to dysfunctional remodeling of joint components via numerous (and frequently associated) etiologies, including mechanical stress, trauma, dental malocclusion, systemic illness, and hormonal and genetic factors [6, 7].

The symptoms of TMJOA are related to an incessant inflammatory process and osteoarthritis (OA). TMJOA symptoms can vary in intensity over time or can be chronic. Chronic orofacial pain is a key symptom of TMJOA and results from persistent synovitis, muscular spasms, and degenerative arthropathy [6]. Other common symptoms are neck pain, joint noises, and limitation of jaw function [8, 9]. Disease severity ranges from mouth-opening limitation to mandibular ankylosis. Pain symptoms seem to be more common and severe for females than males, probably because of hormonal factors [10, 11].

Treatment of TMJOA requires a complex interdisciplinary approach [12]. The diverse therapeutics applied for the management of this polyfactorial disease can be divided into three categories: non-invasive, minimally invasive, and invasive [3]. Non-invasive techniques include physical therapies, occlusal splints, and pharmacologic agents [13]. Manual therapy, therapeutic exercises, and electrophysiological modalities are validated tools to reduce inflammation, relax muscle tension, and improve jaw mobility. Occlusal splints allow the patient to obtain dental occlusion stability, which minimizes pain in the masticatory muscles and joint by holding the bite in the least joint-traumatizing position [14]. They are also useful in case of parafunctional habits such as bruxism, to avoid tooth attrition, malocclusion, and masticatory muscles fibrosis [15]. Nonsteroidal anti-inflammatory drugs (NSAIDs) are commonly prescribed to reduce chronic inflammation but are not without side-effects. Moreover, their short and long-term efficacy are not clearly proven.

Conservative treatment has a real impact [16], but in some cases where noninvasive treatment fails, minimally invasive treatment is applied. Minimally invasive treatment includes intra-articular injections of various substances [e.g., hyaluronic acid (HA)] to enhance the elasticity and viscosity of the synovial fluid and activate tissue repair [17, 18], and NSAIDs and corticosteroids for their anti-inflammatory properties to alleviate pain [19]. Platelet-rich plasma and opioids (e.g., Tramadol^™^ or morphine) can also be employed. Sometimes, intra-articular injections are combined with arthrocentesis to improve therapeutic efficacy [19].

However, minimally invasive therapies have limitations, such as short-term efficacy because of the rapid clearance and degradation of injectable substances. This phenomenon results in the need for repeated injections, which impacts on QoL (e.g. talking, chewing) and healthcare costs [20], and increases the risk of adverse effects. To overcome these problems, researchers are seeking to develop delivery systems that release drugs over a prolonged period after intra-articular injection [20–22]. Our latest systematic review of the literature on this subject [21] revealed that numerous types of drug-delivery system (DDS) have been investigated, such as poly(lactic-*co*-glycolic acid)-based biomaterials [23–25], hydrogels [26], microneedle patches [27], nanostructured lipid carriers [28, 29], and poloxamer micelles [30] as carrier molecules, as well as NSAIDs, HA, and analgesic agents as bioactive molecules. There is a growing interest in the development of such therapeutic methods, and they are being tested *in vitro* and *in vivo* [21].

Among animal models developed for preclinical study of the pathology of TMD or the efficacy of therapeutic methods (e.g., DDSs), the rat is used most often because of its cost and size [31]. Numerous methods are available to induce TMJOA-related pain in rats (e.g., chemical, mechanical, surgical). Our systematic review [32] highlighted that, among these methods, intra-articular injection of chemical agents was used most frequently. The two major chemical agents for injection were complete Freund’s adjuvant (CFA; which is made from inactivated mycobacteria) and monosodium iodoacetate (MIA). Other drugs or agents can also be applied, including collagenase type 2 (Col-2), formalin, and carrageenan [32]. However, the lack of progressive pathological changes led to several drug-induced models being models of cartilage damage rather than OA and, for others, OA-related symptoms lasted only for a few days, which is not suitable for studying the effect of sustained release of a drug or the prolonged effect of a novel DDS. Therefore, a simple and reproducible animal model of TMJOA that mimics the histopathological changes in cartilage and subchondral bone, as well as clinical symptoms (i.e., pain), over a long period (i.e., months), is needed.

In the present study, we compared MIA and Col-2 for their effects in inducing long-lasting TMJOA symptoms in rats. We did not include CFA because of its origin from a mycobacterium, which means that it must be handled under a biosafety hood to prevent contamination. Col-2 was considered because of its well-known pathological effects on other joints (e.g., knee and hip) [33] and therefore its potentially similar effect on TMJs.

MIA is a selective inhibitor of glyceraldehyde-3-phosphate dehydrogenase. Upon administration by intra-articular injection, it provokes persistent degradation and inflammation by disrupting glycolysis, thereby leading to chondrocyte lysis as well as histological and structural modifications of the TMJ [34]. Hence, MIA is used frequently to establish an OA-like animal model [35, 36] for preclinical research. Several studies have shown the reproducibility and reliability of the MIA-induced TMJOA model in rats [36, 37], which has been applied by other research teams to assess the efficacy of their experimental therapies [38, 39]. *Clostridium histolyticum* Col-2 is a mixture of enzymes containing collagenase, non-specific proteases, and clostripain; it acts mainly on collagen, which is known for its regenerative properties [40]. Intra-articular injection of this product has been shown to induce degradation of cartilage and synovial tissue in induction of an OA model in knee joints [33]. Recently, researchers reported their success in inducing TMJOA using Col-2 [41, 42]. However, our systematic review [32] did not reveal a long-term survey of TMJOA induction using this agent. Therefore, there is a lack of substantial data to validate the reliability of Col-2 for the creation of a TMJOA-related model of prolonged pain in rats. Besides, it is difficult to tell whether MIA or Col-2 is more potent for inducing TMJOA because studies using them have differed with regard to the experimental procedures.

The main objective of this study was to compare MIA and Col-2 to select the one that provides the longest and the most reproducible TMJOA-related pain animal model for the study of prolonged drug release from new DDSs.

## Materials and methods

### Ethical approval of the study protocol

Experimental procedures were carried out at the animal facility within the Platform Ressources Expérimentales of the University of Lille (DHURE, Lille, France). The study protocol was in compliance with European rules for the protection of animals used for scientific reasons (Directive 2010/63/EU). Surgical procedures were approved (no. 25897) by the local Animal Experimentation Ethics Committee, as well as the French Ministry of Higher Education, Research and Innovation.

### Animals

Forty male Wistar rats (6-weeks-old; 193–267 g) were divided randomly into two groups of 20. Only adult males were used, to avoid biases related to growth and hormonal factors [38]. Animals were housed in a temperature-controlled room (22 ± 1°C), with a 12-h light–dark cycle. Each rat was placed in an individual cage for ≥7 days. Food and water were available *ad libitum*.

### Chemical induction of TMJOA by intra-articular injection of MIA or Col-2

The injection of MIA or Col-2 into TMJs was applied under an identical protocol. Briefly, under general anesthesia, a solution of the agent was injected into the upper compartment of the left TMJ with a 26-gauge needle following the protocol described by Fuentes and colleagues [43]. To ensure the validity and reliability of this method and to practice our skills, blue dye was used for injection into the TMJs of several rats, and the precision of injection was verified after TMJ dissection.

MIA (0.5 mg; MilliporeSigma, Burlington, MA, USA) was dissolved in 50 μl of physiological (0.9%) saline for injection into the left TMJ of rats in the MIA group. Col-2 (0.2 mg; MilliporeSigma) was dissolved in 50 μl of 0.9% saline for injection into the left TMJ of rats in the Col-2 group. The concentration and volume of injection used referred to the literature [33–35, 44].

**Fig 1:**
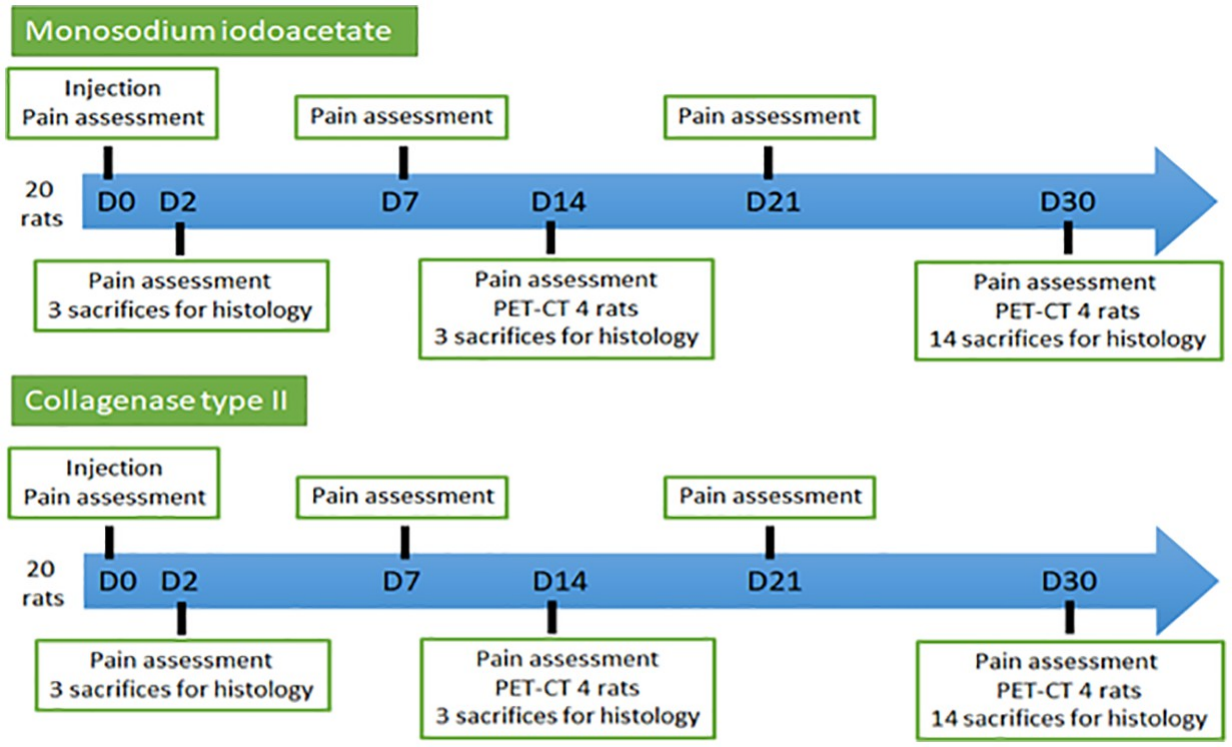
Study protocol.

Three time-dependent components (pain assessment, immunohistological changes, and metabolic function) of TMJ tissues were used to compare the efficacy of MIA and Col-2 in inducing TMJOA. The timeline of the study protocol is summarized in Fig 1.

### Nociception assessment by the Von Frey test

The pain experienced by a rat after injection of a chemical agent into the TMJ was measured by using a Von Frey esthesiometer: this is one of the most popular tests for pain evaluation using animal models. Measurements were made successively before injection of the chemical agent (day 0), then on days 2, 7, 14, 21, and 30 post-injection (Fig 1).

Briefly, before the test, the rat was brought into a quiet room to avoid stress. Then, he was removed from his cage and allowed to move freely for a few minutes. Then, the rat was handled following the guidelines described above for intra-articular injection. A hard-plastic tip was used to stimulate the left-side TMJ. Rat behavior was observed carefully upon increasing the intensity of mechanical stimulation. If head withdrawal or vocalization were observed, the corresponding stimulus intensity (g) was recorded as the head withdrawal threshold (HWT). The HWT was defined as the lowest pressure on the TMJ that induced nociception. Afterwards, the rat was allowed to rest for several minutes. Then, the same manipulation was applied to the right-side TMJ. After each measurement, rats were weighed to monitor their general wellbeing before being returned to their cages.

### Positron emission tomography (PET)

PET was carried out using 2-deoxy-2-[^18^F] fluoro-D-glucose (^18^FDG) as the radiotracer. ^18^FDG-PET is useful for TMD evaluation because of its diagnostic performance for OA and its correlation with the therapeutic response [45, 46]. Imaging was undertaken twice on the same five rats from each group on days 14 and 30. ^18^FDG (150 μl) was injected into the tail vein with a 28-gauge epicranial needle to produce radioactivity of ~40 mBq. Each step was undertaken applying radioprotection measures. The signal intensity of each group is expressed in standard uptake value (SUV).

### Histology

**Fig 2:**
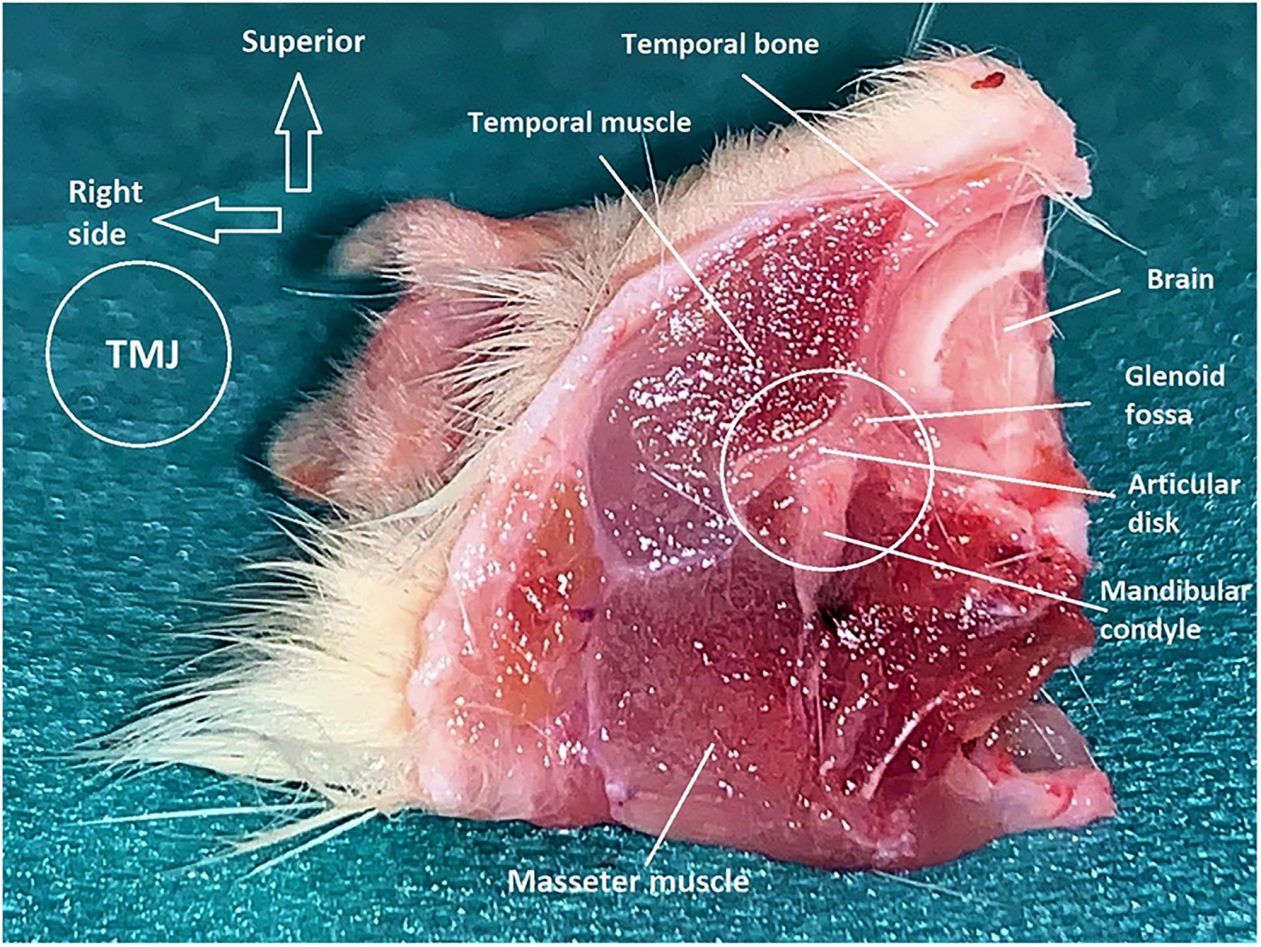
Entire TMJ sample with description.

**Fig 3:**
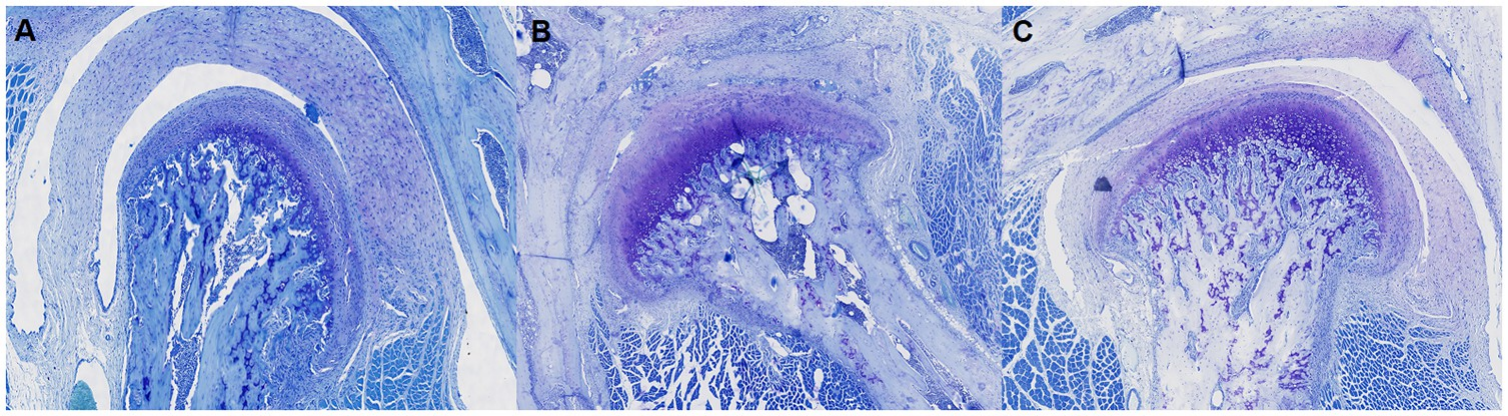
Examples of histological slides after toluidine blue staining. A: sample (before injection of MIA or Col-2) without histological and staining modification; B: sample (day 30 Col-2) with a pannus, hypocellularity, and moderate staining reduction; C: sample (day 30 MIA) with hypocellularity, clefts, and severe reduction of proteoglycan staining.

For each group, on day 2 (*n* = 3) and day 14 (*n* = 3), three rats were selected randomly and killed, and on day 30 all remaining rats (*n* = 14) were killed by intracardiac injection of 0.3 mL of T-61^®^ [embutramide (200 mg/ml), mebenzonium iodide (50 mg/ml), and tetracaine hydrochloride (5 mg/ml); Merck]. Immediately after killing, the whole rat was stored at −80°C until careful dissection of the entire TMJ sample with surrounding tissues (thickness = 5 mm) (Fig 2). Then, explanted samples were fixed immediately in 10% neutral formalin solution at room temperature. After 24 h, samples were decalcified in a 15% ethylenediaminetetraacetic acid solution (pH 7.2) for 4 days, and then stored in 70% ethanol solution at 4°C before undertaking the analytical procedures described below. Each sample was placed separately in a cassette for tissue processing, and dehydrated through a graded series of ethanol solutions (70%, 80%, 90%, 95%, and 100% v/v%), and then embedded in paraffin blocks. A series of sections (thickness = 4–5 μm) was cut using a microtome, and then stained with hematoxylin and eosin and toluidine blue (TB) to highlight morphological changes in articular cartilage in the samples. Stained samples of TMJs were imaged at 10× magnification using a scanner (Axio Scan Z1: Zeiss, Jena, Germany). Fig 3 shows an example of histology sections after TB staining, which allowed precise analyses of the different components of the TMJ.

We wished to assess the degree of TMJOA induced by MIA or Col-2 over time. Cartilage degradation was measured by two observers blinded to the study protocol using a modified version of the Mankin Scale [47, 48]. The latter is used widely in preclinical studies on rat models of TMJOA because of its reliability and ease of use [39, 49]. The Mankin Scale is employed to score cellular and background TB staining, chondrocyte arrangement, and the structural condition of cartilage according to damage severity: a score of 0 is given for normal cartilage and a higher score is allotted for more degenerated cartilage. The final score is the sum of the following items: “structure” (0 to 6); “tidemark integrity” (0 to 1); “proteoglycan staining” (0 to 4); and “cellularity” (0 to 3).

### Statistical analyses

Datasets were analyzed using frequencies or percentages for categorical variables, and mean ± standard deviation (SD) for quantitative variables. Analyses were based on the non-parametric test, Wilcoxon test, or Mann–Whitney test according to the dependence between samples. *p* < 0.05 was considered significant.

With regard to nociception assessment, we first considered each group independently. We compared the mean values of the HWT for the left TMJ at different times (day 0 *vs*. day 2, day 0 *vs*. day 7, day 0 *vs*. day 14, day 0 *vs*. day 21, and day 0 *vs*. day 30), and compared the mean values of the HWT for the TMJ on each side of the rat. Then, we compared the mean values of the HWT for the TMJ on the same side in both groups at different times.

With respect to PET assessment, we compared the mean SUV of each group to that of a control group of rats who did not receive an intra-articular injection. Different times (day 14 *vs*. day 30) in the same group, as well as the two groups at the same time, were also compared, respectively.

## Results

### Clinical observation

For both groups, the bodyweight gain of rats was constant during the 30 days of observation.

In the first hours following intra-articular injection of Col-2 into the left-side TMJs, we noticed hemifacial edema in almost all rats. This hemifacial edema stabilized after a few hours and then regressed in the following days. This complication has not been described in the literature.

### Nociception assessment

To understand the relationship between the nociceptive response and histopathological changes, the HWT was measured at different time points after the injection of MIA or Col-2.

#### Hyperalgesia of TMJs after MIA injection

**Fig 4:**
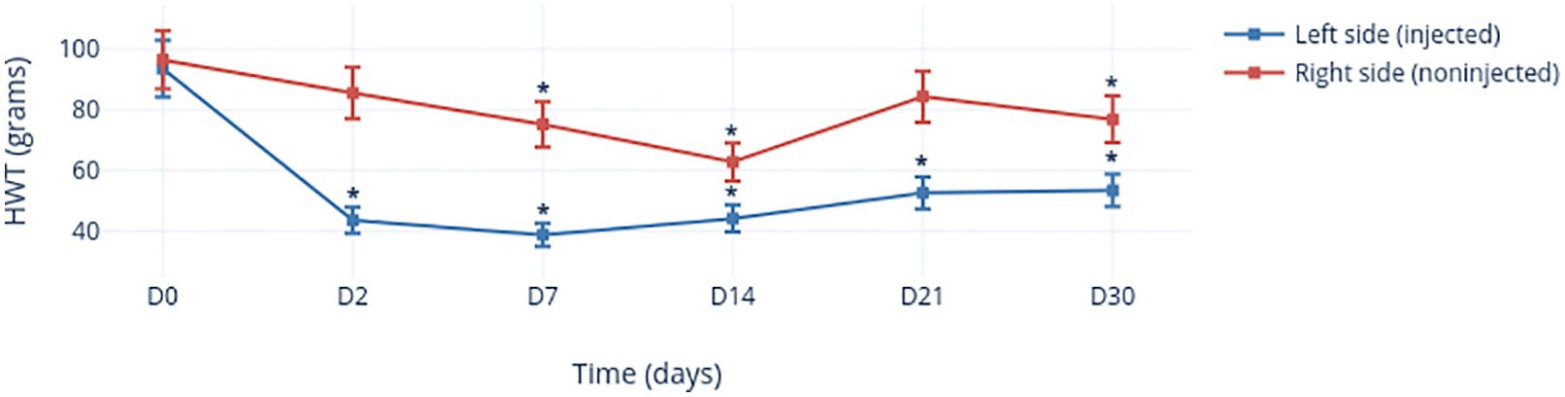
Change in the HWT of TMJs with/without MIA injection as a function of time. *Significant values.

There was no significant difference (*p* = 0.461) between the left TMJ and right TMJ with regard to the HWT before MIA injection (day 0) (Fig 4). The HWT of the MIA-injected side (i.e., the left side) decreased significantly (*p* < 0.0001) 2 days after MIA injection. The HWT decreased significantly until day 30 compared with that at day 0 (*p* < 0.0001). With regard to the right (non-injected) side, a significant decrease in the HWT was found only from day 7 (*p* = 0.017) compared with that at day 0, except for day 21 (*p* = 0.323).

#### Hyperalgesia of TMJs after Col-2 injection

**Fig 5:**
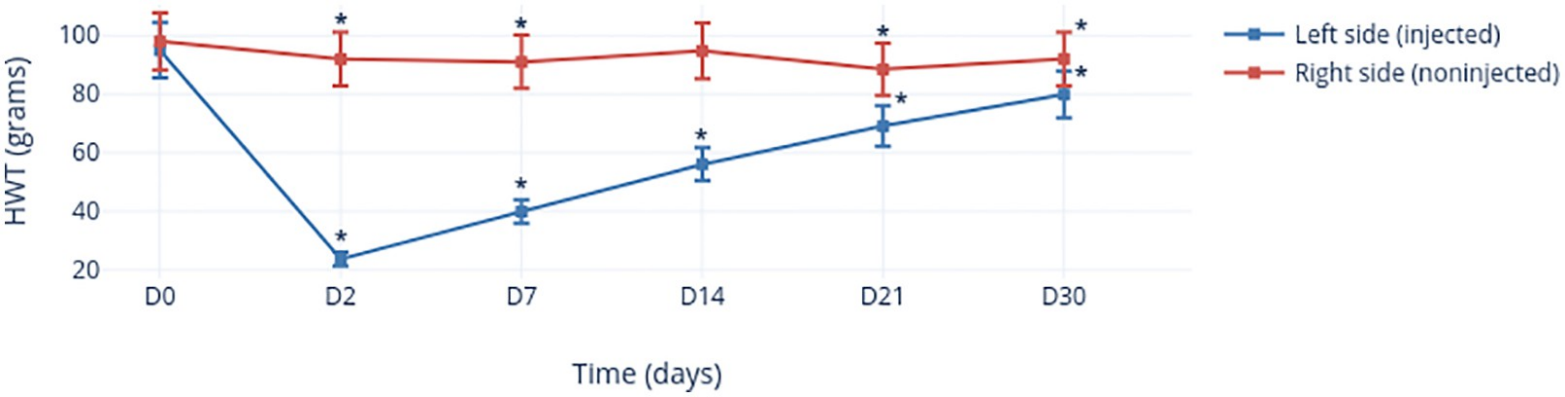
Change in the HWT of TMJs with/without Col-2 injection as a function of time. *Significant values.

There was no significant difference (*p* = 0.277) between the left TMJ and the right TMJ with regard to the HWT before injection of Col-2 (Fig 5). The HWT of the left-side TMJs decreased significantly (*p* < 0.0001) 2 days after Col-2 injection, and then gradually reached the value seen at day 0, while remaining significantly lower than the baseline value at day 30 (*p* = 0.001). With regard to the (non-injected) right side, a significant decrease in the HWT compared with that at day 0 was observed on days 2 (*p* = 0.04), 7 (*p* = 0.036), 21 (*p* = 0.007), and 30 (*p* = 0.036), but not on day 14 (*p* = 0.270).

#### Comparison of the nociceptive response between MIA and Col-2

**Fig 6:**
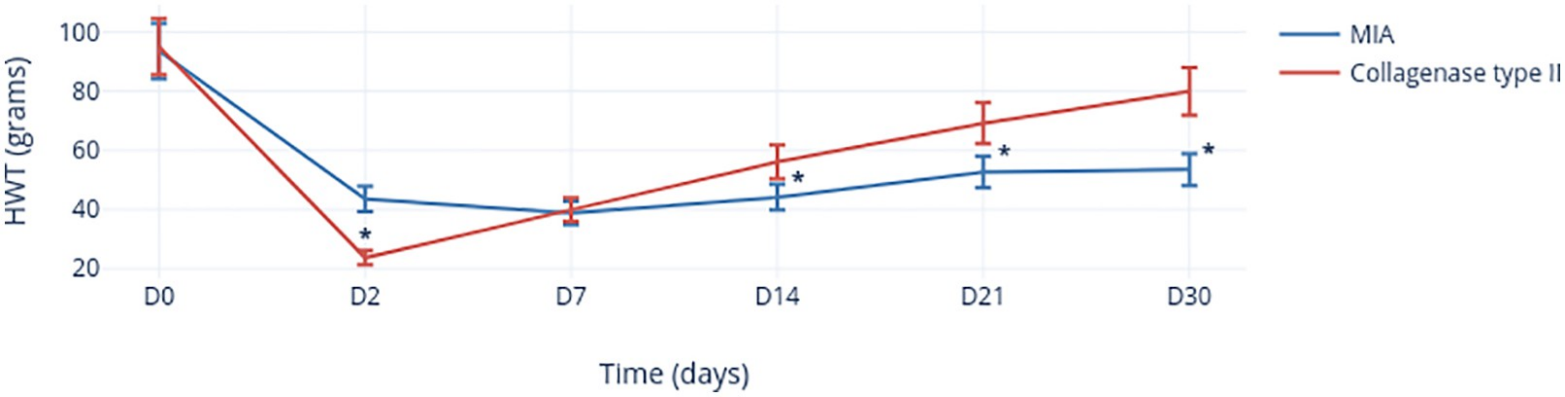
Change in the HWT after MIA (blue) or Col-2 (red) injection as a function of time. Values are the mean ± SD and were analyzed using the Mann–Whitney test. *Significant values.

A comparison of the pain behavior induced by injecting MIA or Col-2 was based on measurements made on the injected (i.e., the left) side (Fig 6). There was no significant difference (*p* = 0.779) in the baseline value (preinjection) of the HWT between the two groups. On post-injection day 2, the HWT of the Col-2 group (23.961 ± 12.136) was significantly lower (*p* < 0.0001) than that of the MIA group (43.585 ± 17.642). No significant difference was found between the two groups at day 7 (*p* = 0.683). However, from day 14 onwards, the HWT of the MIA group was significantly lower than that of the Col-2 group (day 14: 44.161 ± 17.454 *vs*. 56.091 ±16.503, *p* = 0.031; day 21: 52.645 ± 23.454 *vs*. 69.144 ± 8.704, *p* = 0.006; day 30: 53.460 ± 22.537 *vs*. 79.932 ± 10.746, *p* = 0.002).

### Time-dependent histological changes in TMJs

In some specimens, we observed progressive degradation of the different components of the TMJs described in Fig 3. We documented modification of condyle architecture by the appearance of clefts in different cartilage layers or even of geodes in the case of complete disorganization of condyle architecture. Study of the proteoglycan staining present in the cartilage layers highlights cases of moderate or even severe reduction in the uptake of TB dye, indicating an impoverishment of the cartilage structure. A reduction in the number of chondrocytes, leading to hypocellularity, was also noted.

**Fig 7:**
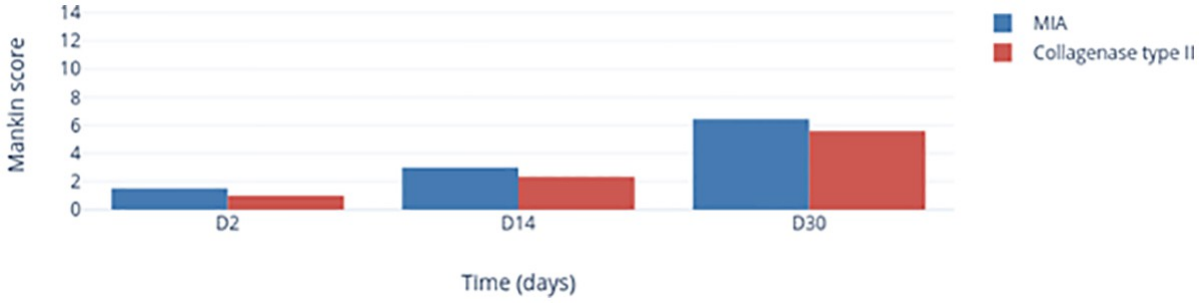
Change in the Mankin Scale score after MIA (blue) or Col-2 (red) injection as a function of time.

For both groups we observed a progressive increase in the Mankin Scale score (MIA group: 1.5 ± 1.5 (day 2) < 3.00 ± 1.00 (day 14) < 6.45 ± 2.86 (day 30); Col-2 group: 1.00 ± 0.67 (day 2) < 2.33 ± 1.56 (day 14) < 5.58 ± 3.68 (day 30) (Fig 7). The Mankin Scale scores of the Col-2 group were slightly lower than those of the MIA group. With regard to the right-side TMJs, the profile was irregular in each group.

### Metabolic changes in TMJs according to PET

**Fig 8:**
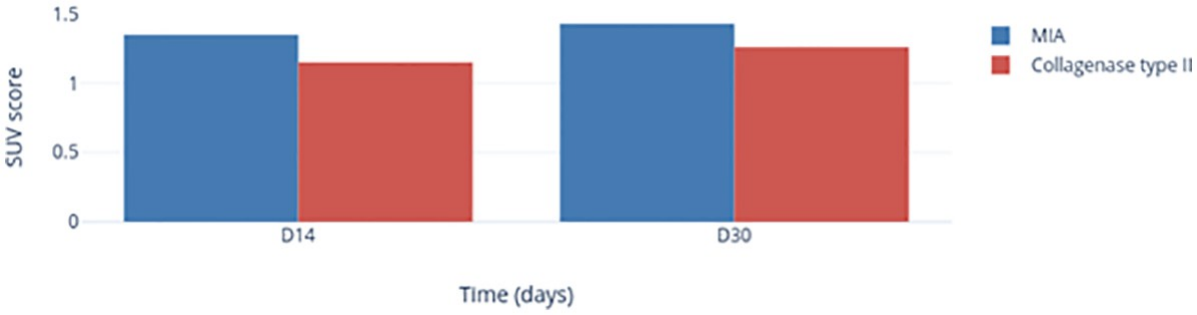
Change in the FDG level after MIA (blue) or Col-2 (red) injection as a function of time.

**Fig 9:**
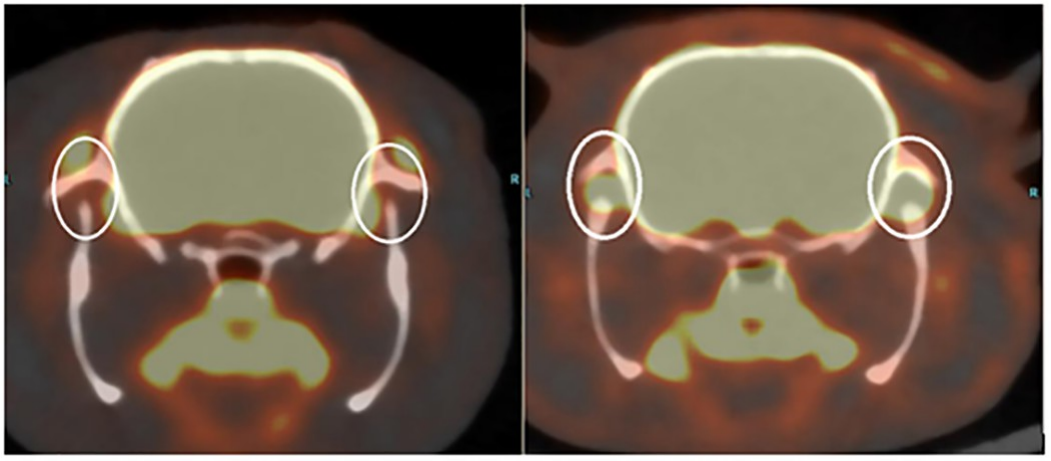
Comparison between signals at TMJs and periarticular muscles in a non-injected rat (left, SUV score = 1.02) and an MIA-injected rat (right, SUV score = 1.91) at day 14.

For the MIA group, we observed a higher SUV score on the left-side TMJs on day 14 (1.63) and day 30 (1.44) compared with that in the control group (1.30) (Fig 8). For the Col-2 group, a similar SUV score was noted on day 14 (1.27) but a lower SUV score was recorded on day 30 (1.15). PET showed a localized hypersignal at TMJs and in periarticular muscles (Fig 9).

## Discussion

Pain is a predominant clinical feature of OA, and may arise from the soft tissues around the TMJ or the subchondral bone undergoing destruction [6]. Therefore, an accurate animal model of OA should have appropriate nociceptive responses corresponding to its histopathological changes. Here, we created a rat model of TMJOA by injection of MIA or Col-2 into the upper compartment of the TMJ. Intra-articular injection of MIA or Col-2 led to time-dependent pain in rats. The clinical expression of chemically induced pain (represented by evolution of the HWT over time) started 2 days post-injection and lasted at least until day 30 post-injection. Such persistent pain is a necessary criterion for validation of a model in studies of sustained-release DDSs. In this context, MIA and Col-2 met this criterion [21, 32]. Our results for MIA injection are in accordance with those in recent studies of an MIA-induced model of TMJOA [37], but our results for the creation of a Col-2-induced model of TMJOA are novel.

The TMJs are the two joints that connect the lower jaw to the skull. The TMJs are paired joints that act together. Unilateral injection of chemicals leads to articular degradation *via* mechanical disturbance on the contralateral side [50]. This was demonstrated in our study: for both MIA and Col-2, the pain threshold of the non-injected side decreased gradually from day 7 until day 30. This pattern of pain evolution could be because TMJOA development takes place in two phases. The first is an inflammatory phase in which acute reaction mechanisms are involved and are resolved early (7–10 days); the second is the product of osteoarticular degradation.

Even if MIA and Col-2 are both effective from a clinical viewpoint, comparison between them allows us to highlight differences in the evolution of the pain-triggering threshold and the intensity of the nociception induced by them. The Col-2 group showed a mean HWT value that was significantly lower than that for the MIA group, similar at day 7, and then higher from day 14 to day 30. These data suggest that Col-2 might have a strong effect initially, but this decreases progressively during the first month. In contrast, MIA maintained a constant action on pain behavior in the TMJOA model. This is an important feature to consider in the choice of agent to create a model for testing a DDS, because the model must be as stable as possible over time to ensure that pain relief is from the prolonged action of the drug and not from a decrease in OA [21, 32].

We must not neglect the early strong adverse effects related to intra-articular injection of Col-2, which were not shown by MIA. These features of Col-2 have not been reported previously. Thus, our results could be a warning against the use of Col-2 for this purpose.

Histology revealed remodeling and degradation of articular components after injection of MIA or Col-2 that was aggravated progressively over time and was maximal at the end of the observation period (day 30). The increase in the Mankin Scale score demonstrated the efficacy of the chemical injection to induce a TMJOA model that led to an acute inflammatory (but also a long-lasting) action. The histological signs of prolonged OA corelated closely with the persistence of lower pain thresholds in nociceptive tests. The nociceptive responses of MIA-induced TMJOA corresponded to histopathological changes. Specifically, TMJ hyperalgesia in the first week after MIA injection could mainly be an inflammatory response whereas, 2–4 weeks after MIA injection, hyperalgesia could be corelated to the subsequent destruction of condylar cartilage and erosion of subchondral bone. When synovitis was alleviated and cartilage damage was repaired by fibrous tissue and subchondral bone underwent a sclerotic change, the nociceptive responses correspondingly returned to those observed at baseline. These data were consistent with known clinical features. For example, patients often experience severe pain during the active–destructive phase of TMJOA with synovitis.

PET is used regularly for the clinical diagnosis and follow-up of TMJ diseases [46]. However, use of PET for a TMJOA model is rare. Hence, our protocol for signal quantification was adapted to use the data fully. Use of PET provided interesting data for study of our TMJOA models. PET allows precise visualization of marked inflammatory phenomena in small animals. PET showed a localized hypersignal at the TMJs and in periarticular muscles (Fig 9) that was a reaction to the MIA injection; this effect lasted ≥30 days. There was a lack of change in the SUV score in the Col-2 group, a feature which favors use of MIA to establish a TMJOA model.

Therefore, overall, MIA appeared to be more suitable than Col-2 for creating a rat model of TMJOA-related pain for the study of prolonged drug release. The constant character of the pain, which lasted ≥30 days, is the principal reason for this conclusion, because pain is the principal symptom of TMJOA.

This is the first study proposing a protocol for creation of a rat model for study of a DDS [32]. The long-term (30-day) nature of our study allowed creation of a reproducible model for study of DDSs. Another advantage was the combination of clinical, histological, immunohistochemical, and PET data to study different aspects (pain and OA) of this complex disease, which can be used to verify the global action of a DDS.

Our study had one main limitation, related mostly to technical difficulties encountered during animal manipulation. The SD values of the HWT in the MIA group tended to be greater than those for the Col-2 group. This finding may imply a progressive improvement of our skills in handling of the rats. Therefore, a longer learning curve for animal handlers than expected may be necessary.

## Conclusions

MIA and Col-2 induced orofacial pain by their local chemical action on TMJs. However, based on its prolonged and greater sustained effect on the pain threshold, persistent histological changes and imaging results, MIA appeared to be more suitable than Col-2 for creation of a rat model of TMJOA. Use of MIA produces a long-lasting TMJOA-related pain animal model for the study of prolonged drug release from new DDSs. This model will also be valuable for other teams because of its ease of use and reproducibility.
